# Temporal variation in climatic factors influences phenotypic diversity of *Trochulus* land snails

**DOI:** 10.1038/s41598-022-16638-w

**Published:** 2022-07-19

**Authors:** Małgorzata Proćków, Elżbieta Kuźnik-Kowalska, Aleksandra Żeromska, Paweł Mackiewicz

**Affiliations:** 1 Museum of Natural History, University of Wrocław, Sienkiewicza 21, 50-335 Wrocław, Poland; 2 Department of Bioinformatics and Genomics, Faculty of Biotechnology, University of Wrocław, Joliot-Curie 14a, 50-383 Wrocław, Poland; 3 Department of Invertebrate Systematics and Ecology, Institute of Environmental Biology, Wrocław University of Environmental and Life Sciences, Kożuchowska 5b, 51-631 Wrocław, Poland

**Keywords:** Ecology, Evolution, Zoology

## Abstract

Organisms with limited dispersal capabilities should show phenotypic plasticity in situ to keep pace with environmental changes. Therefore, to study the influence of environmental variation on the phenotypic diversity, we chose land snails, *Trochulus hispidus* and *T. sericeus*, characterized by high population variability. We performed long-term field studies as well as laboratory and common garden experiments, which revealed that temporal environmental changes generate visible variation in shell size and shape of these snails. Many shell measurements of *T. hispidus* varied significantly with temperature and humidity in individual years. According to this, the first generation of *T. hispidus*, bred in controlled laboratory conditions, became significantly different in higher spire and narrower umbilicus from its wild parents. Interestingly, offspring produced by this generation and transplanted to wild conditions returned to the ‘wild’ flat and wide-umbilicated shell shape. Moreover, initially different species *T. hispidus* and *T. sericeus* transferred into common environment conditions revealed rapid and convergent shell modifications within one generation. Such morphological flexibility and high genetic variation can be evolutionarily favored, when the environment is heterogeneous in time. The impact of climate change on the shell morphometry can lead to incorrect taxonomic classification or delimitation of artificial taxa in land snails. These findings have also important implications in the context of changing climate and environment.

## Introduction

The phenotypic variation of populations is essential for natural selection to drive evolution. However, the importance of evolutionary processes responsible for this variation is still unclear^1^. Phenotypes are generated by the interaction of genetic factors, e.g., mutations, gene flow and drift, with epigenetic variation, phenotypic plasticity, maternal effects and various environmental factors^2–7^. Individual genomes determine unique phenotypes, which develop under specific environmental conditions. However, the genotype and the environmental influence would not effectively drive evolution without developmental plasticity, i.e., the ability of producing phenotypic variants by the same genotype depending on the environment^8^.

The influence of environmental factors on phenotypes is difficult to investigate due to the large number of possible interactions. However, land snails, whose shell morphology can change under environmental factors, are ideal organisms to study the phenotypic evolution due to their low mobility and specific habitat requirements^9,10^. Their phenotypic plasticity can be associated with habitat^11,12^, growth season duration^13,14^ and environment pollution^15^. Many surveys focused on intraspecific variation in qualitative features, e.g., the color and pattern of shells of terrestrial snails^7,16–18^, but quantitative variation was insufficiently studied. Strong associations between intraspecific variation in body size and organism’s environment were usually detected in space^13,19–21^ but temporal differentiation was poorly explored^22–24^. Constraints on the season duration strongly affect the final body size especially in short-lived taxa with an annual life cycle because their growth can occur only over several months.

A good model for investigations of phenotypic variation in a short temporal scale can be the hairy snail *Trochulus hispidus* (Linnaeus, 1758). It is the most widespread and abundant species of its genus, with high plasticity in shell size and shape revealed under laboratory conditions^25^. It is considered a species complex^26^ consisting of populations of at least two ecophenotypes, *T. hispidus* and *T. sericeus*^27^. They cannot be regarded as biological species because they do not form distinct gene pools and have no reproductive barriers. Therefore, successful cross-breeding is possible between them^25^. Their conchological differences are limited to absolute and relative umbilicus diameter^28,29^. These euryoecious snails exhibit a predominantly annual life cycle^30,31^. Previous studies showed that the shell size and shape of wild *T. hispidus* changed significantly within one generation and the new morphology also persisted during the second generation of snails bred in laboratory conditions different from that in the wild environment^25^. High inter-individual variance in lifetime fecundity, fertility and survival of *T. hispidus* was also observed in the laboratory^31^ but its general life cycle does not seem to depend on the geographic location in England and Poland^30,32^. *Trochulus hispidus* snails, characterized by a short life span, a high reproductive output and semelparity, seem well adapted to a locally unpredictable environment^31^ and a wide range of habitats^33^.

In order to assess to which extent and magnitude environmental components can influence phenotypic plasticity, we studied populations of land snails, *T. hispidus* and *T. sericeus*. Changes in their shell size and shape were recorded in wild populations during 1 year, taking into account fluctuating local climatic conditions year-to-year. Additionally, common garden and controlled laboratory experiments were carried out to assess the influence of new environmental pressures on shell morphometry. Taking into account the high phenotypic variability of these snails, we can expect that the changes in shell size and shape are associated with climatic and environmental changes.

## Results

### Temporal differentiation of wild populations of *T. hispidus* and climatic parameters

**Figure 1:**
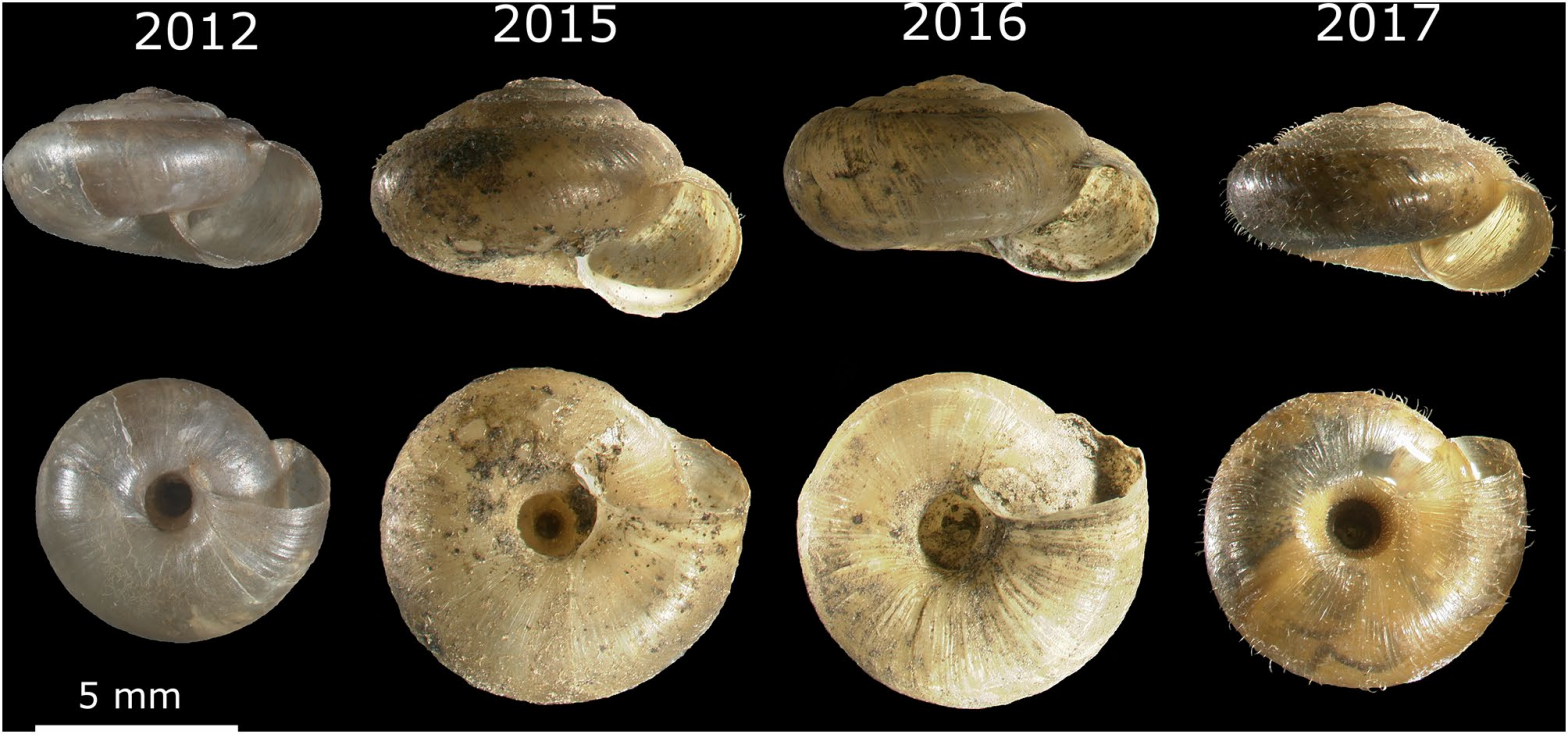
Shells of *Trochulus hispidus* collected in different years in Wrocław.

**Figure 2:**
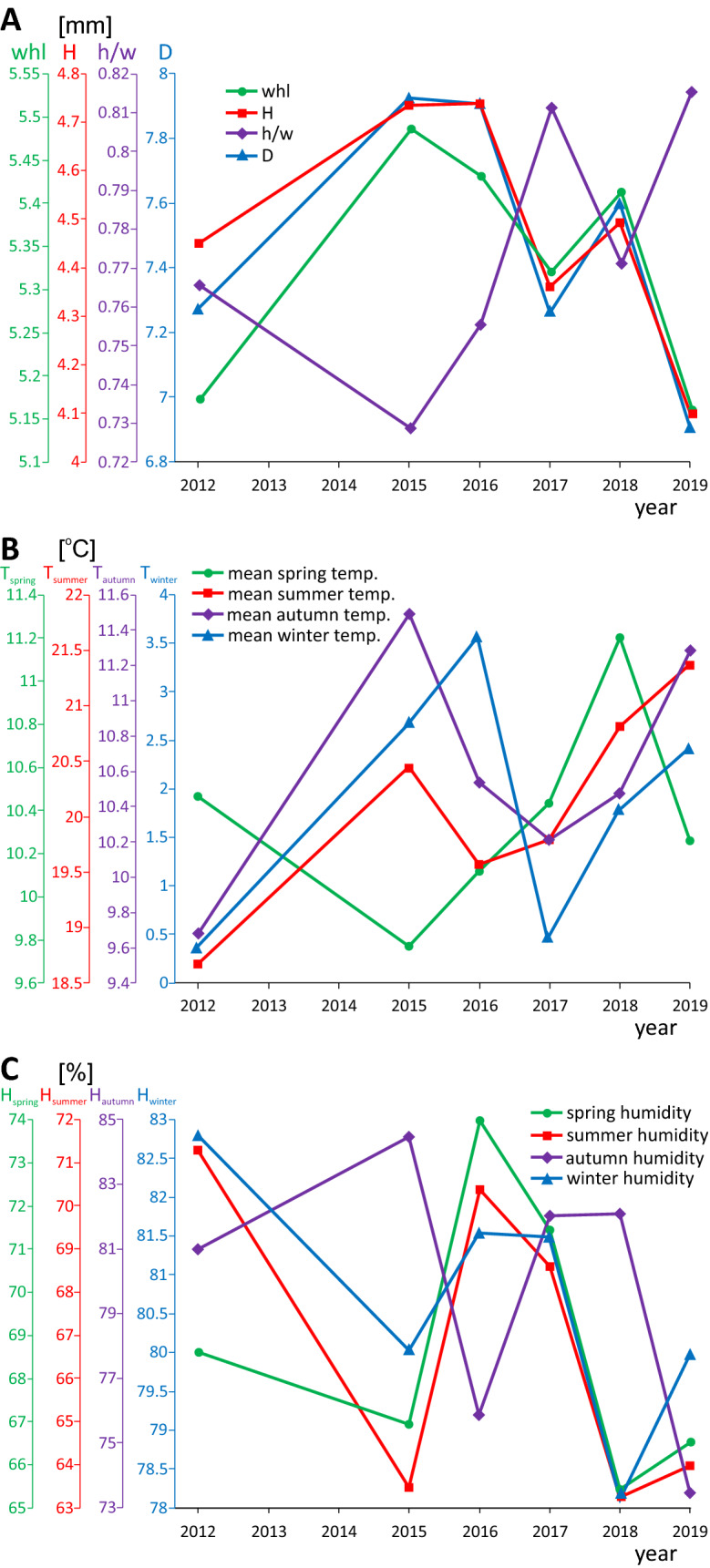
Changes in: mean values of selected morphometric features of shells collected in various years in Wrocław (**A**) as well as the mean temperature (**B**) and the relative humidity (**C**) recorded in four seasons in Wrocław in eight-year period. Abbreviations: D—shell diameter (in mm), H—shell height (in mm), h/w—aperture height/width ratio, whl—number of whorls. The summary statistics for A is included in Table S1 and original data in Table S10 in Additional file 2.

Comparison of morphometric features of *T. hispidus* shells collected in different years in two geographic regions, i.e., Wrocław and Lubawka, showed significant differences depending on the year of collection. The largest number of differences was revealed in shells from Wrocław (Figs. 1 and 2A; Additional file 2: Table S1). Out of 210 comparisons (15 pairs of collection years × 14 features), 84 were statistically significant (Additional file 2: Table S2), e.g., shell diameter (D) was significantly different in 11 cases, shell height (H) and shell width (W) in 10 cases, body whorl height (bwH), the number of whorls (whl), umbilicus major (U) and minor (u) diameters in 9 cases and aperture height/width ratio (h/w) in 7 cases. Nine features obtained more than 10% difference between shells in at least one comparison of mean values, e.g., U 24%, u 19%, H 16% and D 15% (Additional file 2: Table S2). Umbilicus major (U) and minor (u) diameters showed the largest average percentage difference, i.e., 12% and 10%, respectively, in comparisons of all years.

For snails from Lubawka, out of 84 comparisons (6 pairs of collection years × 14 features) only 8 were statistically significant (Additional file 2: Table S3). The shells differed significantly in their aperture height (h) and width (w) in 3 comparisons. The h feature showed the percentage difference up to 9% (Additional file 2: Table S3) and the largest average difference was 4.5%.

Besides the phenotypic variation, climatic parameters also showed high fluctuations in the studied period (Fig. 2B,C, Additional file 2: Table S4). The maximum difference reported between temperature parameters in some years prior to sample collection in Wrocław was up to 3.7 °C for the maximum winter temperature, while the maximum difference in the relative humidity was up to 11% for autumn. The maximum temperature difference in Jelenia Góra close to Lubawka was up to 3.5 °C for the minimum winter temperature, while the relative humidity differed at most by up to 8% in summer.

### Differences in shell morphometry under various climatic conditions

**Figure 3:**
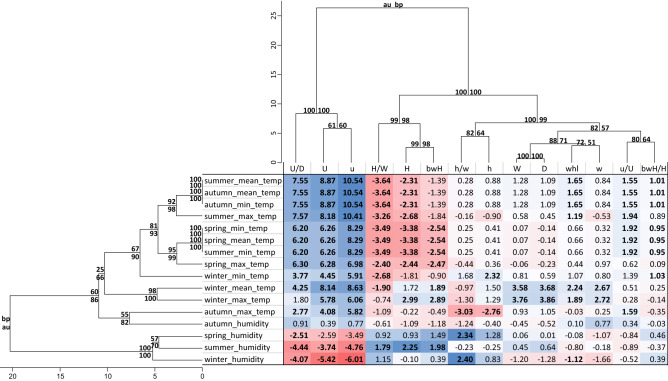
Mean percentage differences in morphometric features between shells that were grown in different conditions. The shells were divided into two groups according to the average value of a given climatic parameter for each season and studied region. The first group included the shells that developed in conditions above this average and the second below this average. Positive values indicate that the given feature was greater in the first group, whereas negative values indicate that this feature was greater in the second group. Dendrograms cluster the features and the parameters according to their similarity in the percentage differences. Values marked in bold indicate statistically significant differences between the compared groups of shells. Values at the dendrogram nodes indicate significance assessed according to approximately unbiased test (au) and bootstrap resampling (bp).

The distinction between shells collected in individual years and changes in climatic parameters along the same period suggest that these differences can be associated with the climate. Therefore, we calculated the average value of a given climatic parameter for each season and studied region and next divided the collected shell data into two groups according to this value. The first group included the shells that developed in conditions above this average and the second below this average (Additional file 2: Table S5). The differences between these groups were statistically significant for 15 out of 16 considered climatic parameters for at least two shell features (Fig. 3). Similarly, each of 14 features significantly separated the groups based on at least two climatic conditions. The results demonstrated that the mean winter temperature substantially influenced nine morphometric shell features, whereas eight characters were changed due to the maximum winter temperature as well as the mean and minimum temperatures in spring, summer and autumn. Umbilicus major (U) and minor (u) diameters as well as umbilicus relative diameter (U/D) were significantly different in 14 pairs of groups characterized by various climatic parameters. In 11 pairs, the height/width ratio (H/W) was significantly different and shell height (H) in 10 pairs.

The umbilicus diameters (u and U) as well as umbilicus relative diameter (U/D) clustered together in the dendrogram based on the mean percentage difference, which indicates that they similarly responded to climatic conditions (Fig. 3). The features u and U revealed the strongest average increase of all features, from 4.1 to 10.5% in shells developed in higher temperatures in all seasons. The largest percentage difference exceeding 10% was recorded for groups separated according to the mean summer and autumn temperatures as well as the maximum summer and minimum autumn temperatures. The U/D ratio was also significantly greater with the mean percentage difference of 2.8–7.6% in shells grown under high temperatures for all seasons and almost all temperature types. On the other hand, the u and U diameters as well as the U/D ratio were on average by 3.7–6.0% significantly smaller in shells developed under higher humidity in summer and winter.

The height/width shell ratio (H/W) was grouped with H and bwH features in the dendrogram and was on average by up to 3.6% significantly smaller in shells grown under higher temperatures in all seasons for almost all types of parameters. The maximum winter temperature caused a significant increase, on average by ca. 3%, in shell height (H) and body whorl height (bwH), whereas higher temperatures in other seasons led to their decrease by up to 3.4% (Fig. 3).

The shells that were grown in autumn with a relatively high maximum temperature were characterized by ca. 3% significantly smaller aperture height (h) and aperture height/width ratio (h/w), which were clustered together in the dendrogram (Fig. 3).

Other four features, shell diameter (D), number of whorls (whl) as well as shell (W) and aperture width (w), formed an additional cluster in the dendrogram (Fig. 3). All of them were on average significantly greater in shells collected one year after winter that was characterized by relatively higher mean and maximum temperatures. The percentage difference was greater, with 3.6–3.9% for W and D.

In the dendrogram, the climatic parameters were clustered in several groups indicating their similar influence on the morphometric features of shells (Fig. 3). There are separate clusters for temperature and humidity parameters with the exception of the autumn maximum temperature and autumn humidity, which are grouped together. The other temperature parameters for warmer seasons are separated from those for winter, which indicates that they differently influenced the shell morphometry.

### Correlations between morphometric shell features and climatic parameters

**Figure 4:**
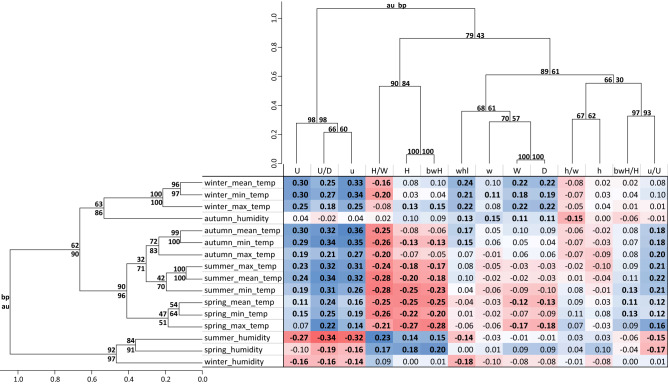
Spearman’s correlation coefficients between morphometric features of shells with climatic parameters under which the snails were grown. Dendrograms cluster the features and the parameters according to their similarity in the coefficients. Values marked in bold are statistically significant. Values at the dendrogram nodes indicate significance assessed according to approximately unbiased test (au) and bootstrap resampling (bp).

The influence of climatic conditions on the shells collected in individual years was also assessed using Spearman’s correlation coefficient between the morphometric features and climatic parameters (Fig. 4). Of 224 potential relationships 113 were statistically significant. The spring mean temperature was significantly correlated with 10 morphometric features. Summer humidity and six temperature parameters, i.e., the minimum temperatures as well as the spring and winter maximum temperatures, significantly correlated with eight shell features. Minor umbilicus diameter (u) and umbilicus relative diameter (U/D) were significantly correlated with almost all climatic parameters, i.e., 15, umbilicus major diameter (U) and height/width ratio (H/W) with 13 and the ratio of umbilicus minor to its major diameter (u/U) with 11.

As in the case of percentage difference, we can also recognize groups of morphometric features that were similarly correlated with climatic parameters (Fig. 4). Features U/D, U and u were significantly positively correlated with all or almost all temperature parameters for four seasons with the coefficients up to 0.34, 0.30 and 0.36, respectively. On the other hand, the significant correlation coefficients between these features and the humidity in spring, summer and winter were negative and reached − 0.34.

Another group of features included shell height/width ratio (H/W), shell height (H) and body whorl height (bwH) (Fig. 4). All of them showed significant negative correlations with all temperature parameters for spring and summer as well as the minimum autumn temperature, and H/W also with the mean and maximum autumn temperatures as well as the mean and minimum winter temperatures. The correlation coefficients reached − 0.28, − 0.27 and − 0.28, respectively. These three features significantly correlated with summer and spring humidity, at up to 0.23.

The number of whorls (whl), shell width (W), shell diameter (D), demonstrated a similar correlation with climatic parameters (Fig. 4). They showed the largest and significant correlation coefficients with winter temperatures: up to 0.24, 0.22 and 0.22, respectively. The ratio of umbilicus minor to its major diameter (u/U) showed significant positive correlation up to 0.22 with temperature of warmer seasons.

The climatic parameters were grouped into several clusters indicating their similar relationships with morphometric features (Fig. 4). Humidity parameters of warmer seasons formed a separate cluster and temperature parameters were grouped according to seasons. The winter parameters were connected with autumn humidity and separated from temperatures for warmer seasons.

### Modelling relationships between morphometric shell features and climatic parameters

The joint influence of many climatic parameters on morphometry of shells collected in individual years was studied using a linear mixed-effects (LME) model after exclusion of correlated parameters and a linear ridge regression (LRR) model including all climatic parameters. The latter allows for the inclusion of correlated variables. We separately investigated the seasonal maximum, mean and minimum temperature parameters in combination with seasonal humidity parameters (Additional file 2: Table S6) because they are obviously correlated.

Umbilicus minor (u) and major (U) diameters as well as umbilicus relative diameter (U/D) turned out best explained by the climatic parameters (with R^2^ > 0.15) in two models (Additional file 2: Table S6). Moreover, u, U and U/D were described in LME models by the largest number of significant climatic parameters, i.e., 15. The features u and U had also the largest number of significant parameters in LRR models, i.e., 18 out of 24 possibilities. The largest average values of temperature coefficients for the LRR models were 0.66 for D, 0.58 for W, 0.32 for H, 0.26 for U and 0.22 for u. Thus, all the above-mentioned features were under the strongest influence of the climatic conditions.

In the case of LRR models, the coefficients at the winter mean temperature were most often selected as significant, in 12 out of 14 possibilities (Additional file 2: Table S6). The humidity coefficients for autumn were significant in 30 cases of 42 possibilities. The highest average absolute values of coefficients in climatic variables were those for the summer (0.63), spring (0.31) and autumn (0.24) minimum temperatures as well as the summer mean temperature (0.31). Thus, the temperatures of warmer seasons were more important for developing shell morphology. Seasonal humidity coefficients showed similar values compared to each other.

### Comparison of shell morphometry of *T. hispidus* and *T. sericeus* kept under various conditions

**Figure 5:**
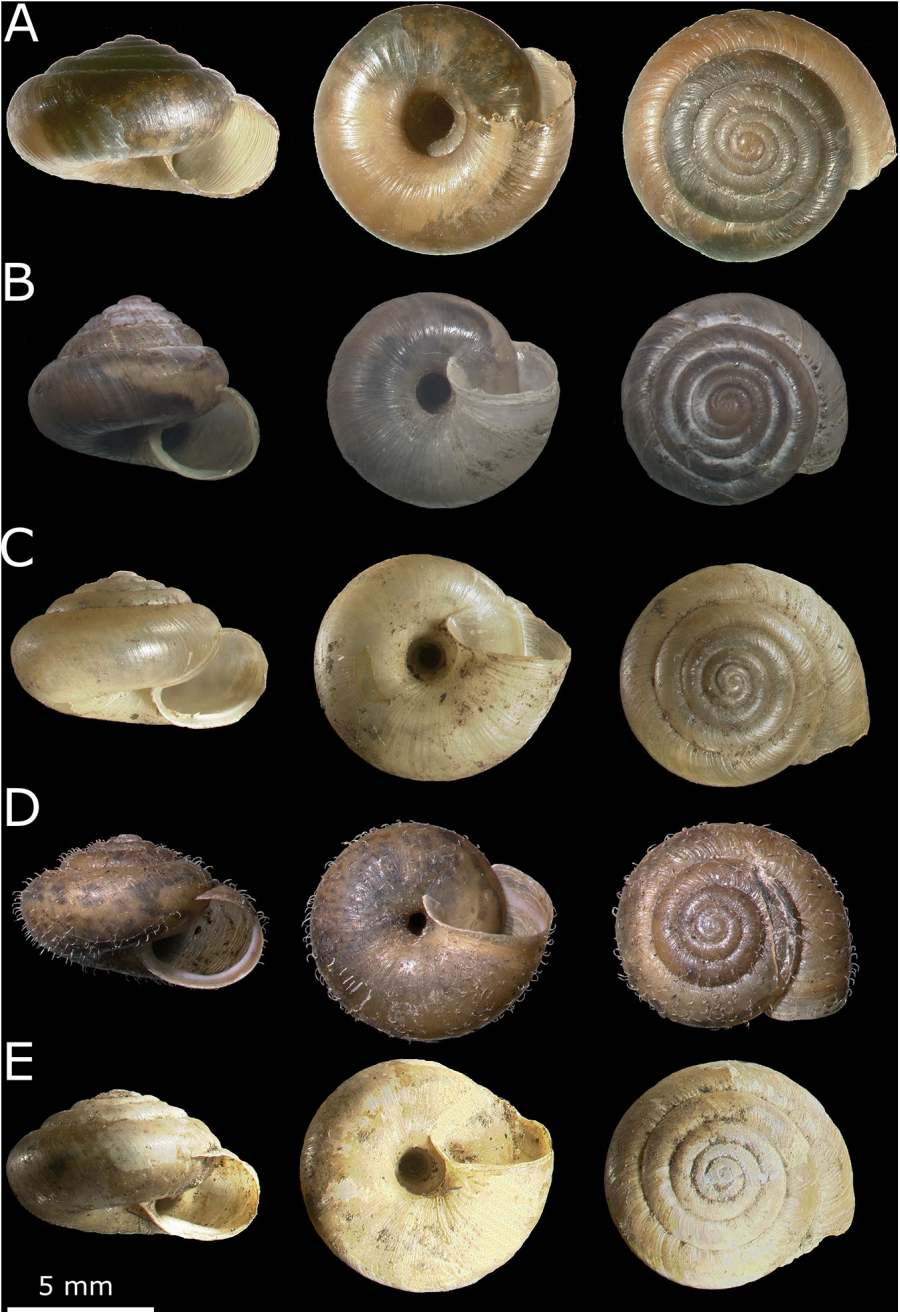
Shells of two *Trochulus* ecophenotypes: parental *T. hispidus* from wild habitat in Wrocław (**A**), the first generation of *T. hispidus* raised in laboratory (**B**); *T. hispidus* reared in garden in Wrocław (**C**); *T. sericeus* from wild habitat in Muszkowice (**D**); *T. sericeus* reared in garden in Wrocław (**E**).

In order to verify the influence of different climatic parameters on *Trochulus* shell morphometry in selected conditions, we compared shells from three groups of *T. hispidus*, which represented several subsequent generations: (1) parental snails collected in the wild in Wrocław-Jarnołtów, (2) their offspring bred in the laboratory for two generations and (3) offspring of the second laboratory-bred generation transplanted again into a garden in Wrocław (Fig. 5A–C). The comparison of the group 2 and 1 was to verify if laboratory conditions with controlled temperature and humidity can influence the shell morphometry within only one generation, whereas including the group 3 in the comparison, we wanted to check if snails raised in wild garden conditions can recover the original phenotype. Furthermore, we transplanted into the same garden conditions *T. sericeus*, which was collected in the wild in Muszkowice (Fig. 5D,E). In this case, we verified if two originally different ecophenotypes *T. hispidus* and *T. sericeus*, develop the same shell morphometry under the same conditions.

Conditions in which these snails developed were different. According to WorldClim, the wild environment of *T. hispidus* in Wrocław was generally warmer than that of *T. sericeus* in Muszkowice (Additional file 2: Table S7). The largest difference was 1.4 °C for the maximum summer temperature. Relative humidity was lower in Wrocław by up to 2% for warmer seasons but was higher in winter by 1.6%. The difference between the wild and garden localities in Wrocław was much smaller and did not exceed 0.41 °C. The garden conditions were less humid, by up to 2%. However, data from WorldClim are generalized over a longer period and wider regions, so may not well reflect local conditions in the studied places. Actually, the Wrocław site was an open habitat covered with a nettle community like a garden patch, while the Muszkowice site was overgrown by a beech forest, which most likely maintained a higher humidity and a more stable temperature.

Laboratory temperatures were substantially different from those in the field, especially for winter (by 18–19.7 °C) as well as for spring and autumn (by 8.2–12 °C). Laboratory humidity was by up to 4.5% lower compared to winter and 5.9–9.9% higher than in spring and summer.

**Figure 6:**
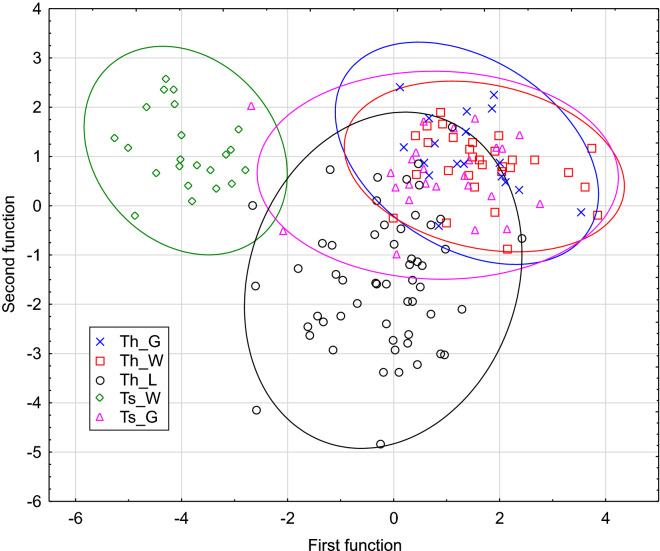
Discriminant function analysis plot for five groups of *Trochulus*: parental *T. hispidus* from wild habitat in Wrocław (Th_W), the first generation of *T. hispidus* raised in laboratory (Th_L); *T. hispidus* bred in garden in Wrocław (Th_G); *T. sericeus* from wild habitat in Muszkowice (Ts_W); *T. sericeus* bred in garden in Wrocław (Ts_G).

A discriminant function analysis (DFA) for the defined groups of snails provided their interesting grouping and separation (Fig. 6). The analysis identified three significant discriminant functions (*p* < 00002). The first two functions explained 63% and 27% of variance, respectively. The first function was best positively correlated with umbilicus relative diameter (U/D, r = 0.82) as well as umbilicus major and minor diameters (U and u, r = 0.63). The second function was best positively correlated with relative height of body whorl (bwH/H, r = 0.43) and negatively with shell height (H, r = − 0.68), shell diameter (D, r = − 0.51), shell width (W, r = − 0.50), aperture width (w, r = − 0.48), body whorl height (bwH, r = − 0.47) and aperture height (h, r = − 0.43).

In the DFA plot (Fig. 6), the wild *T. sericeus* created a distinct group separate from others. The laboratory-bred *T. hispidus* also formed a well-defined cluster, which only partially overlapped the other sets. Many laboratory-kept specimens were located far from others in the plot, which indicates their disparate shell morphometry. However, wild *T. hispidus* as well as garden *T. sericeus* and *T. hispidus* were grouped together.

**Figure 7:**
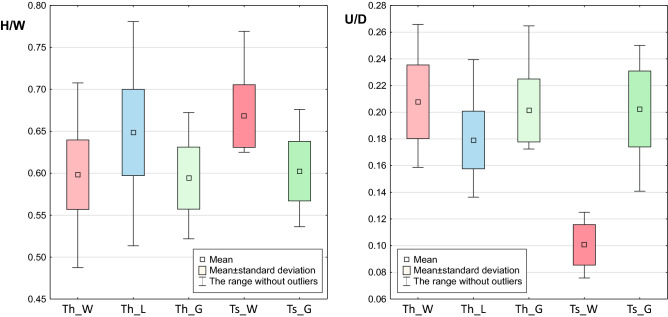
Boxplots of selected morphometric features, height/width ratio (H/W) and umbilicus relative diameter (U/D), for five groups of *Trochulus*: parental *T. hispidus* from wild habitat in Wrocław (Th_W), the first generation of *T. hispidus* bred in laboratory (Th_L); *T. hispidus* raised in garden in Wrocław (Th_G); *T. sericeus* from wild habitat in Muszkowice (Ts_W); *T. sericeus* raised in garden in Wrocław (Ts_G).

In agreement with DFA, statistical tests showed that the laboratory-bred *T. hispidus* differed significantly in 9 and 10 morphometric features from the wild parents of *T. hispidus* and its garden-bred offspring, respectively (Additional file 2: Tables S8 and S9). The average percentage differences calculated from the absolute values of all individual features were in these cases 7.3% and 9.8%, respectively. The wild and garden *T. hispidus* appeared to be different in 6 features with an average percentage difference of 6.4%. Aperture width (w) and height/width ratio (H/W) increased significantly by 10% and 8% in the laboratory snails in comparison to their wild parents (Fig. 7). Then, after their offspring were raised in the garden, these features decreased significantly by 17% and 9% to the values that were not statistically significantly different from those of the wild parents. The same trend was followed by height of body whorl (bwH) as well as shell height (H), width (W) and diameter (D). The initial increase in these features was 14–5% and the subsequent decrease was 23–13%. The garden-raised *T. hispidus* had the shell statistically different from the wild forms, but the laboratory snails were still much more distinct in these features among the compared groups. On the other hand, umbilicus relative diameter (U/D) and relative height of body whorl (bwH/H) decreased by 16% and 5% in the laboratory-bred *T. hispidus* in comparison to their wild parents, and then became larger by 12% and 4% in the garden snails, which were not statistically different form the wild *T. hispidus* in these features (Fig. 7).

The wild *T. sericeus* was significantly different from the wild *T. hispidus* in nine features with an average percentage difference of 28% and differed in six features from its descendants raised in the garden on average by 26% (Additional file 2: Tables S8 and S9). The latter differed only in 4% in four shell features from *T. hispidus* which lived in the same conditions. U/D, U and u were the most distinctive differences between *T. sericeus* and *T. hispidus* from the wild environment (Fig. 7). These features were smaller in the former by 115–107% and were subjected to a substantial average increase by 110–101% in *T. sericeus* raised in the garden. After this drastic change, the offspring became statistically indistinguishable from *T. hispidus* living in the same conditions. The same trend was observed in the number of whorls (whl), which was significantly smaller in the wild *T. sericeus* than in the wild *T. hispidus* by 6%. After the garden experiment, whl increased significantly by 4% and the shell became similar to that of the wild *T. hispidus*. In turn, height/width ratio (H/W) was larger in the wild *T. sericeus* than in *T. hispidus* by 12%, and dropped by 11% in the garden snails to the value observed in the wild *T. hispidus* (Fig. 7).

### Correlation between climatic parameters and shell morphometry of *T. hispidus* and *T. sericeus* kept under various conditions

We noticed significant correlations between the selected morphometric features and the climatic parameters. For example, the maximum summer temperature was positively correlated with umbilicus relative diameter (U/D) with the Spearman’s correlation coefficient of 0.64, while negatively correlated with height/width ratio (H/W), body whorl height (bwH), shell height (H) and aperture width (w). The correlation coefficients were − 0.56, − 0.55, − 0.53 and − 0.47, respectively. The relative humidity in spring and summer showed positive relationships with shell height (H, ρ = 0.65), body whorl height (bwH, ρ = 0.56), aperture width (w, ρ = 0.54), shell height/width ratio (H/W, ρ = 0.47), and aperture height (h, ρ = 0.47). Shell height (H) was also positively correlated with the minimum autumn temperature (H, ρ = 0.47) and negatively with the winter humidity (H, ρ = − 0.47). Relative height of body whorl (bwH/H) demonstrated the highest positive correlations with autumn (bwH/H, ρ = 0.49) and winter humidity (bwH/H, ρ = 0.48), and negative (bwH/H, ρ = − 0.48 and − 0.49) with almost all temperature parameters except the maximum summer temperature.

## Discussion

This and previous studies^21,25,27^ show that *T. hispidus* and its congener *T. striolatus* are excellent models for investigating phenotypic evolution in response to changing environmental conditions. Here we found that the temporarily varying environment is important in shaping phenotypic variation. Our investigations not only confirm a great morphological variation within *T. hispidus*^34,35^ but also demonstrate that year-to-year changes in the local climate generate temporal variation within its populations. The influence of climatic parameters was clearly demonstrated in laboratory-bred snails that differed significantly from their parents. Furthermore, we revealed rapid and convergent shell modifications of initially different *T. hispidus* and *T. sericeus* transplanted to a new environment. Our findings show that phenotypic differences do not have to be genetically determined, but can be environmentally induced and manifested in response to even small environmental variation^19,36,37^ and controlled laboratory conditions^25,38–41^.

Weldon^42^ and Di Cesnola^43^ studied variation of early whorls in shells from land snails belonging to Clausiliidae and Helicidae. They noticed a smaller variation in shell whorls of adults than in immatures and concluded that it is the example of stabilizing selection. Hutchinson^44^ critically commented these results and among several reasons proposed that this different variation can result from that the whorls in adults and immatures were formed in different years or seasons. This explanation agrees with our findings. Weldon^42^ and Di Cesnola^43^ also claimed that the mean shape of these whorls was the same in adult and immature shells but we found significant differences in mean values of many adult shell features. Thus, our results would indicate rather a directional selection. However, these features were variable in time and could change even within one generation, so they were not genetically inherited.

The observed phenotypic changes can be associated with differentiated mechanisms of shell growth and production in the specific climatic conditions. The greatest sensitivity to climate parameters was demonstrated for absolute and relative umbilicus diameters (u, U and U/D). These features increased with higher temperatures in all seasons and decreased with higher humidity in spring, summer and winter. Umbilicus diameters as well as shell width and height were also extremely variable within populations of *T. striolatus* and showed negative correlations with precipitation^21^. In this study, lower temperatures and associated higher humidity most likely positively influenced the growth of whorls and their greater tightness around the columella of the *T. hispidus* shell. The less tightly coiled whorls can be associated with their larger number (whl) in higher winter and autumn temperatures. Accordingly, shells of gastropods existing in winters with milder and higher temperatures were wider in terms of width (W) and diameter (D) because they could continue their growth in warmer conditions. Alternatively, a selection could operate against smaller shells during harsh cold winters in the temperate climate when snails hibernate. Depending on the varying temporal conditions, uneven growth around the columella determining u and U (Additional file 1: Fig. S1) was reflected by positive and negative correlations of u/U with spring, summer and autumn temperatures, respectively as well as spring and summer humidity.

An opposite influence of temperatures and humidity was observed on features associated with height, i.e., shell height (H), height/width ratio (H/W) and height of body whorl (bwH). This suggests an adaptive advantage of higher and more globular shells in more humid and cooler conditions mostly in spring and summer, in which the shell growth is the most intense^20,45^. The surface-to-volume ratio of globular shells seems to be more beneficial to face dehydration in high temperature conditions^46^. This smaller ratio could also help survive in colder conditions. Larger shell apertures (h and w) as well as relative height of body whorl (bwH/H) are developed in higher humidity. However, under water stress conditions, a small aperture, which decreases the area of exposed surface, as well as a flat shell, which allows snails to penetrate deeper into vegetation or under stones, could be advantages in more dry conditions^13,47^.

The size of adult gastropods is determined by the growth rate, which may be influenced by food supply or temperature^48^. In the Mediterranean region, a slowdown in growth rate is caused by heat and drought^49,50^ and cessation of growth may occasionally occur in snails aestivating during periods with drought in a temperate climate^14,45^. We found significant differences in *T. hispidus* shell size correlated with ambient temperature and humidity. The great phenotypic plasticity may be adaptive and enable various shell growth patterns under variable environmental conditions^41^. The high plasticity may increase the population persistence but reduce the likelihood of genetic change, because the plastic response itself places the population close to an adaptative peak^51^.

*Trochulus hispidus* is an euryoecious species, which may spread and occupy places differing greatly in environmental conditions both spatially and seasonally in the same place. The great variation of this species was reflected not only in its plastic morphology manifested by two ecophenotypes, *T. hispidus* and *T. sericeus*^27^, but also in a bet-hedging mode of life. In particular, fluctuating water and temperature regimes were supposed to influence its variable mortality, which may account for short-lived and fast-reproducing strategy of this species^31^. This variation was also shown at the genetic level and the presence of highly divergent phylogenetic clades among *T. hispidus*^26^. This plasticity may evolve in response to changes in environmental variability^52,53^. In the case of *T. hispidus*, it could be in the Pleistocene as a response to the variable climate with alternations of cold and warm periods.

In the light of our findings, the capacity for evolutionary and plastic responses appeared to be high in *T. hispidus*, which corresponds to a general conclusion that species with shorter generation times, higher initial population sizes and higher fecundity are expected to evolve faster for a given absolute rate of physical change^54^. Moreover, organisms with limited dispersal capabilities such as land snails can keep pace with environmental changes only by adaptation in situ.

The growth of snails can be potentially limited by food availability. However, the food shortage may be excluded as an important limiting factor of *Trochulus* growth in the wild environment because *T. hispidus* shows plasticity in its feeding habits, regardless of variation in available sources^30^. These snails are small in size and often live in the litter with various food resources, so the conditions in which the collected snails lived most probably did not limit the availability of food. Moreover, laboratory as well as field and common garden experiments ensured trouble-free access of food. Other factors (competitors or predators) were also under control. If the food availability were the determining factor, we should have observed the largest variation in shell height and width. In contrast to that, the shape expressed by ratios and umbilicus diameters showed the greatest changes.

This and our former studies^27^ show that plasticity is evolutionarily favored when the environment is heterogeneous in time and space. Selection promotes various phenotypes in different environments because no single phenotype has the greatest fitness across all environments^52,55–57^. On the other hand, the evolution of phenotypic plasticity can be substantially constrained by costs associated with acquiring environmental information, producing different phenotypes and maintaining the physiological and developmental capacity for facultative responses^52,58,59^.

With its plastic feeding habits, flexible life-history tactics as well as morphological and genetic variation^26,27,30,31^, *T. hispidus* perfectly matches theoretical models, which prove that the more variable populations are associated with broader niches and distribution ranges, decreased intraspecific competition, extinction risk, vulnerability to environmental changes and fluctuations in population size but increased productivity, growth rate, establishment success, invasiveness and speciation rate (reviewed by^60,61^). The *Trochulus* snails could be a good indicator of climate changes proceeding both on local and global scales as well as in shorter and longer terms. The temperature effect was greater than humidity and large enough to detect the increasing climatic warming.

## Conclusions

Our long-term field studies as well as laboratory and common garden experiments revealed that seasonal changes in local climate generate substantial variation in the shell size and shape of land snails of the genus *Trochulus*. The shell measurements fluctuated according to changes in temperature and humidity in individual years. The first generation of *T. hispidus* bred in a laboratory differed significantly from its wild parents and reversed after moving to field conditions. Initially different *T. hispidus* and *T. sericeus* transplanted to a common environment also revealed rapid and convergent shell modifications in a short time. The results provide evidence that plasticity is evolutionarily favored when the environment is heterogeneous in time and space. This visible influence of climate on shell morphology can lead to incorrect taxonomic classifications or the delimitation of artificial taxa. The studied snails could be a good indicator of climate changes that occur both on local and global scales as well as in shorter and longer terms. Our results show that rapidly changing climate and environment, e.g., due to human activity, can be reflected in morphometry of small invertebrates such as gastropods.

## Methods

### Sampling, biometric measurements

We collected 411 *T. hispidus* adults during 4–6 growing seasons in four localities in Poland: two in Wrocław and two in Lubawka (Table 1). Eight measurements were taken from the adult shells using a calibrated eyepiece in stereomicroscope with accuracy 0.001 mm (Additional file 1: Fig. S1): shell height (H), shell width (W), body whorl height (bwH), aperture height (h), aperture width (w), umbilicus major diameter (U) (i.e., the longest diameter parallel to the shell diameter, D), umbilicus minor diameter (u) (i.e., perpendicular to umbilicus major diameter) and shell diameter (D). The number of whorls (whl) was counted according to^62^ (Additional file 1: Fig. S1). The specimens were measured once by the same person (MP) in the standardized views (Additional file 1: Fig. S1;^33^). Moreover, the following coefficients of shell proportions were calculated: height/width ratio (H/W), relative height of body whorl = body whorl height/shell height ratio (bwH/H), umbilicus relative diameter = umbilicus major diameter/shell diameter ratio (U/D), ratio of umbilicus minor to its major diameter (u/U), aperture height/width ratio (h/w). Shell measurements and their ratios for individual specimens were included in Additional file 2: Table S10.

**Table 1:** Characteristics of sampling localities of *T. hispidus.*

Locality	Wrocław—Jarnołtów (WJ)	Wrocław—Las Pilczycki (WLP)	Lubawka 1 (L1)	Lubawka 2 (L2)
Coordinates	51° 07′ 16.9″ N 16° 50′ 38.7″ E	51° 08′ 59.8″ N 16° 57′ 25.9″ E	50° 42′ 19.2″ N 16° 00′ 09.8″ E	50° 41′ 58.1″ N 15° 59′ 59.8″ E
Altitude a.s.l	130 m	112 m	420 m	505 m
Habitat type	Lowland	Lowland	Submontane zone	Submontane zone
Plant community	Mainly *Urtica dioica*, single *Fraxinus excelsior* and *Ulmus laevis* trees	Trees of *Salix fragilis* and *Carpinus betulus*; undergrowth: *Urtica dioica*, *Aegopodium podagraria, Galium aparine*	Nettle (*Urtica dioica*) patch with a single tree of *Acer platanoides* and shrubs of *Symphoricarpos albus*	Nettle (*Urtica dioica*) patch
Canopy	30%	70%	30%	0%
Herb layer	70%	70%	70%	70%
Length of growing season*	226 days	226 days	188 days	188 days
Collecting date (no. of shells)	21.06.2012 (30)			
	14.11.2015 (27)	14.11.2015 (30)		
	22.10.2016 (25)	22.10.2016 (16)	01.10.2016 (20)	01.10.2016 (22)
	15.10.2017 (24)	15.10.2017 (20)	07.10.2017 (20)	07.10.2017 (31)
	07.10.2018 (23)	07.10.2018 (20)	01.10.2018 (26)	01.10.2018 (23)
	22.09.2019 (6)	22.09.2019 (8)	29.09.2019 (20)	29.09.2019 (20)

*Data from^66,67^.

### Laboratory and common garden experiments

**Figure 8:**
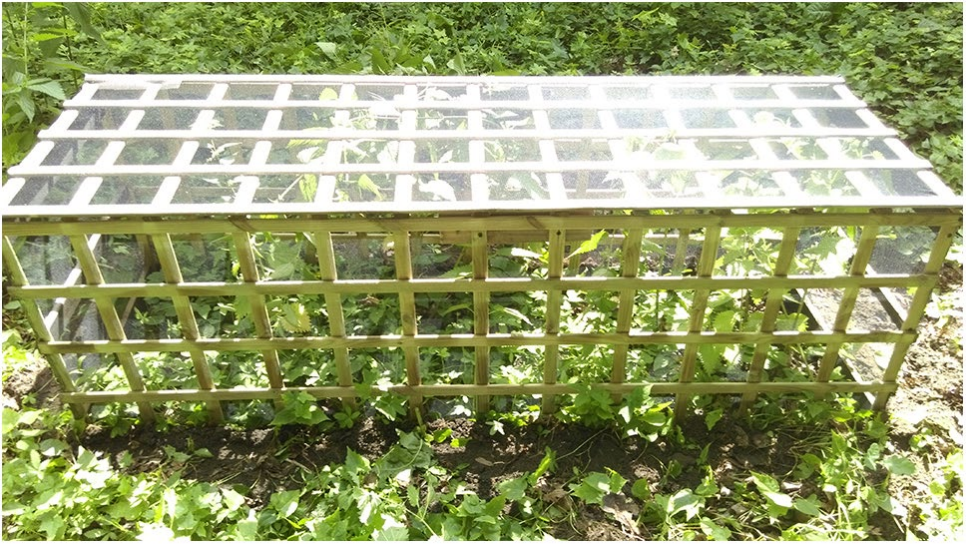
A snail cage made of wooden slats covered with a dense mesh net used in the experiment. The mesh size is ca. 1.5 mm to prevent snails escape.

Laboratory *T. hispidus* snails, whose parents were collected in the wild in Wrocław-Jarnołtów, were bred in light/dark 12/12 photoperiod at 22 °C and 15 °C, respectively, as well as 80% relative humidity^25^. For the common garden experiment, we used 25 adults and 39 subadults of the second generation of laboratory-bred. These snails were released in a nettle patch limited by a cage with the size 180 × 62 × 60 cm (Fig. 8), which is large enough for this species considering its seasonal variation of density of 84–369 individuals/m^2^^30^. Additionally, 160 eggs laid by the F_2_ generation were implanted. All shells were marked with nail polish to distinguish the transplanted parents from their offspring (Additional file 1: Fig. S2). The experiment lasted from June 2016 to November 2017 in a garden in Wrocław (51° 05′ 54.2″ N 17° 05′ 41.2″ E), the city from which some laboratory-bred snails originated. All the experiments ensured trouble-free access of food and other factors, such as competitors or predators were eliminated. An analogous experiment was carried out with *T. sericeus* ecophenotype. Altogether 199 snails with shells consisting of 3.0 to 5.5 whorls, collected in 14.05.2018 and 04.07.2019 from Muszkowice (50° 38.458′ N 16° 57.05′ E), were transplanted to a nettle patch bounded by the cage identical to that of *T. hispidus*. The experiment lasted from May 2018 to May 2020.

Prior to the experiments, adult snails with 5 and more whorls out of 25 laboratory-bred *T. hispidus* and 22 field-collected *T. sericeus* were measured. At the end of the experiments all empty shells and live snails were collected and adult offspring were measured, including 18 *T. hispidus* and 25 *T. sericeus*. Shell measurements and their ratios for individual specimens of *Trochulus* snails from the wild, laboratory experiment and garden experiment were included in Additional file 2: Table S11. The shell morphometry of the snails raised in the garden was compared with that of the snails originally collected in the wild environment and the laboratory-bred *T. hispidus*.

### Climatic data

We included easily available climatic data that can influence land snail growth and distribution, i.e., temperature and humidity. We used humidity rather than precipitation because the former parameter is more important for snail growth, richness and abundance^63,64^. It influences the snails more directly than precipitation.

The climate data for the studies with snails collected in Wrocław and Lubawka in various years were obtained from two meteorological stations of the Institute of Meteorology and Water Management—National Research Institute (IMGW-PIB), Wrocław and Jelenia Góra, located closest to the sampling locality, i.e., 6 km for Wrocław-Jarnołtów (WJ) and 10 km for Wrocław-Las Pilczycki (WLP) as well as 40 km for Lubawka (L1 and L2), respectively. We collected monthly averages of: (1) mean daily temperature; (2) maximum daily temperature; (3) minimum daily temperature and (4) mean daily relative humidity. To reduce the number of parameters, we did not study the data for each month but we calculated arithmetic means of these parameters for autumn (September, October, November), winter (December, January, February), spring (March, April, May), and summer (June, July, August) for each sampling year (Additional file 2: Table S4). Therefore, we included 16 climatic parameters. Since the studied snails reached maturity and the final body size after ca. 12 months^31^, the climate data from 12 months preceding the date of shell collection were used for comparison with the shell morphometric data.

In the study of snails involved in the common garden experiments, the climatic parameters, that is saturation vapor pressure, mean, maximum and minimum temperature for four seasons, were obtained from WorldClim database (https://www.worldclim.org) with a resolution of 30 s for regions from which *Trochulus* snails were collected for culture in selected conditions: Wrocław-Jarnołtów, a garden in Wrocław and Muszkowice (Additional file 2: Table S7). Relative humidity was calculated based on the formula by^65^ using saturation vapor pressure and appropriate temperature values.

### Statistical analyses

Appropriate statistical tests were performed to evaluate the significance of differences between shell morphometric features for snails classified in various groups according to the year and season of their collection for two geographic regions (Wrocław and Lubawka), the ecophenotypes (*T. hispidus* and *T. sericeus*) and the breeding conditions, i.e., wild environment, laboratory and garden. The tests were also applied for shells divided into two groups, including samples collected one year after the value of the given climatic parameter in the region was greater or smaller than the average. The average was calculated for the minimum and maximum values of the given parameter reported in the two regions studied, Wrocław and Lubawka. We also calculated the percentage difference between the mean values of the shell morphometric features of the compared groups.

In the statistical analyzes, we applied the Shapiro–Wilk test to verify if the analyzed variables followed a normal distribution. The homogeneity of variance in the analyzed groups was checked using the Lévene test. Two groups were compared using unpaired Welch’s t-test if the assumption on the normality of distribution was fulfilled. Otherwise, its non-parametric counterpart, i.e., the unpaired Wilcoxon–Mann–Whitney test, was applied. In the comparison of more than two groups, we used the parametric one-way Analysis of Variance (ANOVA) with moderately conserved Tukey HSD post-hoc test, when the assumptions about normality of distribution and variance homogeneity were fulfilled. When at least one of these assumptions was violated, we applied the non-parametric counterpart of ANOVA, Kruskal–Wallis test with Dunn's post-hoc test in pairwise comparisons between groups.

Spearman’s correlation coefficients (ρ) and their significance were calculated between individual shell morphometric features and climatic parameters for *T. hispidus* collected in different years in Wrocław and Lubawka as well as *T. hispidus* and *T. sericeus* kept under various conditions. We applied the Benjamini–Hochberg method for *p* value correction to control the false discovery rate in the post-hoc tests and when many hypotheses were tested, e.g., correlations between shell morphometric features with climatic parameters as well as differences between two groups of shells developed in conditions characterized by a value of a given climatic parameter greater or smaller than the average reported in the studied regions. Differences were considered significant when *p* value was less than 0.05.

Morphometric features and climatic parameters were subjected to hierarchical clustering based on the percentage differences between snails developing in different climatic conditions and the correlation coefficients. The dendrograms were constructed using UPGMA (unweighted pair group method with arithmetic mean) and Euclidean distances. To evaluate the reliability of individual clades in the obtained dendrograms we applied the approximately unbiased tests and bootstrap resampling assuming 1000 replications.

To model relationships of shell morphometric features with many climatic parameters, we applied a linear mixed-effects model in lmer function in R package lme4. Sites (localities) of collected samples were assumed as random-effects. To avoid correlations between the climatic parameters (seasonal humidity as well as maximum, mean and minimum temperatures), we applied the variance inflation factor (VIF) and removed variables with VIF exceeding 10. Moreover, we applied a linear ridge regression model to include all climatic parameters using lmridge function in R package lmridge. Appropriate ridge biasing parameter K was selected for each case individually based on the minimum mean square error (MSE). Due to a high correlation between seasonal maximum, mean and minimum temperature parameters, we included them separately in combination with seasonal relative humidity. Discriminant Function Analysis (DFA) including Canonical Analysis (CA) was carried out on all morphometric features for five groups of shells: *T. hispidus* from wild, laboratory and garden conditions, as well as *T. sericeus* from wild and garden environments.

The statistical analyses were performed in R software 3.5 (A language and environment for statistical computing, R Core Team 2019, R Foundation for Statistical Computing, Vienna, Austria) and Statistica (data analysis software system, version 13, TIBCO Software Inc. 2017). We used the following R packages: stats, car, FSA, lme4, fmsb, pvclust and lmridge. Shell measurements and their ratios for individual specimens were included in Additional file 2: Tables S10 and S11.

### Ethics statement

Samples were taken only on communal lands. No specific permission was required for crossing these areas or carrying out snail surveys. The target species are not protected by any national law or local regulations.

## Supplementary Information

Supplementary Information 1.

Supplementary Information 2.

Supplementary Information 3.

## Data Availability

All data generated or analysed during this study are included in this published article and its supplementary information files.
