# Supplementary material for: Temporal variation in climatic factors influences phenotypic diversity of Trochulus land snails
Source: Sci Rep. 2022 Jul 19;12:12357. doi: 10.1038/s41598-022-16638-w (PMC9296580; doi:10.1038/s41598-022-16638-w)

Figure S1. Scheme of shell measurements used in the study; frontal view A: height (H), width (W), body whorl height (bwH), aperture height (h), aperture width (w); bottom view B: umbilicus major diameter (U), umbilicus minor diameter (u) and shell diameter (D); C: whorls' counting.

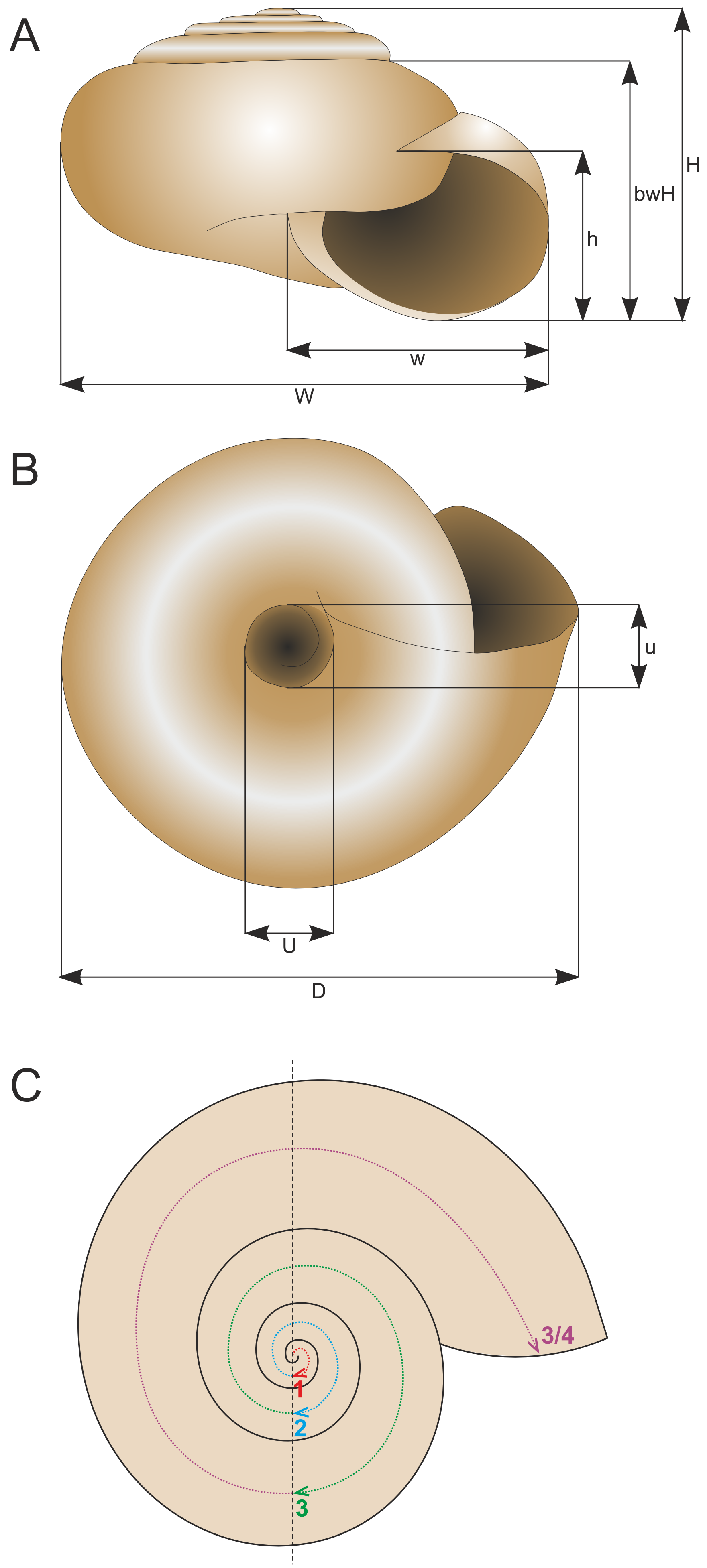

Figure S2. Transplanted *Trochulus sericeus* parent marked with red nail polish (A) and its adult offspring (B).

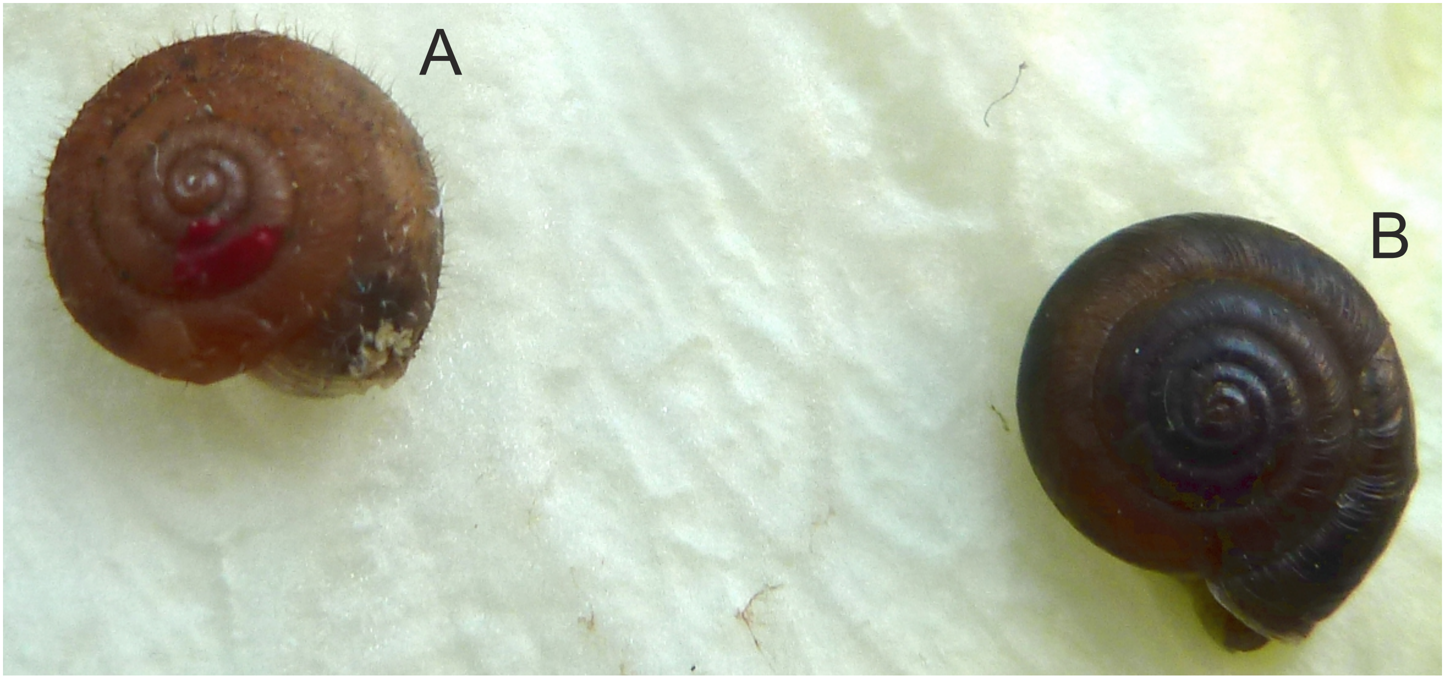

Supplement: Supplementary file 1 — Supplementary Information 1. [file 41598_2022_16638_MOESM1_ESM.pdf]
