# Supplementary material for: Temporal variation in climatic factors influences phenotypic diversity of Trochulus land snails
Source: Sci Rep. 2022 Jul 19;12:12357. doi: 10.1038/s41598-022-16638-w (PMC9296580; doi:10.1038/s41598-022-16638-w)
Supplement: Supplementary file 3 — Supplementary Information 3. [file 41598_2022_16638_MOESM3_ESM.docx]

**Supplementary Information**

**Additional file 1:**

**Figure S1.** Scheme of shell measurements used in the study; frontal view A: height (H), width (W), body whorl height (bwH), aperture height (h), aperture width (w); bottom view B: umbilicus major diameter (U), umbilicus minor diameter (u) and shell diameter (D); C: whorls’ counting

**Figure S2.** Transplanted *Trochulus sericeus* parent marked with red nail polish (A) and its adult offspring (B)

**Additional file 2:**

**Table S1.** Basic statistics of morphometric features of shells collected in different regions and years

**Table S2**. Percentage difference between morphometric features of shells collected in different years in Wrocław. Statistically significant differences were marked in bold

**Table S3.** Percentage difference between morphometric features of shells collected in different years in Lubawka. Statistically significant differences were marked in bold

**Table S4.** Climatic parameters for two regions and studied period used in the study

**Table S5.** Basic statistics of morphometric features in two groups of shells developed in conditions characterized by a value of a given climatic parameter greater (>) or smaller (<) than an average reported in the studied regions

**Table S6.** Characteristics of models describing relationships between shell features and climatic parameters

**Table S7.** Climatic parameters for regions from which *Trochulus* snails were collected to culture in selected conditions. Data were obtained from WorldClim database

**Table S8.** Basic statistics of *Trochulus* snails from: wild environment (W), laboratory experiment (L) and garden experiment (G)

**Table S9.** Mean percentage differences in morphometric features between *Trochulus* shells that were grown in selected conditions

**Table S10.** Shell measurements in mm and their ratios for individual specimens collected in different regions and years

**Table S11.** Shell measurements in mm and their ratios for individual specimens of *Trochulus* snails from natural environment, laboratory experiment and garden experiment
